# Effect of switching basal insulin regimen to degludec on quality of life in Japanese patients with type 1 and type 2 diabetes mellitus

**DOI:** 10.1186/s40780-015-0027-2

**Published:** 2015-09-30

**Authors:** Morihiro Okada, Masae Okada, Jun Nishigami, Naoto Yamaaki, Kenji Furukawa, Kiminori Ohyama, Tsutomu Shimada, Yoshimichi Sai

**Affiliations:** Department of Pharmacy, Japan Community Healthcare Organization Kanazawa Hospital, Ha-15 Oki-machi, Kanazawa, 920-8610 Japan; Department of Medicinal Informatics, Graduate School of Medical Sciences, Kanazawa University, 13-1 Takara-machi, Kanazawa, 920-8641 Japan; Department of Internal Medicine, Japan Community Healthcare Organization Kanazawa Hospital, Ha-15 Oki-machi, Kanazawa, 920-8610 Japan; Department of Hospital Pharmacy, University Hospital, Kanazawa University, 13-1 Takara-machi, Kanazawa, 920-8641 Japan

**Keywords:** Degludec, Glargine, Detemir, HbA1c, DTR-QOL, QOL, BOT

## Abstract

**Background:**

Maintainance of a stable basal insulin level is important for glycemic control in treatment of diabetes mellitus. Recently introduced insulin degludec has the longest duration of action among basal insulin formulations. The purpose of this study was to assess changes in quality of life (QOL) associated with switching the basal insulin regimen to degludec in patients with type 1 and type 2 diabetes mellitus.

**Methods:**

This 24-week open-label intervention study included type 1 (*n* = 10) and type 2 (*n* = 20) diabetes mellitus patients, with adequately controlled hemoglobin A1c (HbA1c), who had received insulin glargine or detemir for at least 6 months. The primary outcome was change of QOL from baseline, as assessed by the Diabetes Therapy-Related QOL (DTR-QOL) application, after switching from glargine or detemir to degludec. HbA1c and other parameters were also assessed as secondary outcomes.

**Results:**

QOL and HbA1c in patients with type 1 diabetes mellitus were unchanged during this study. In patients with type 2 diabetes mellitus, HbA1c did not change, but total DTR-QOL score was significantly improved from baseline after switching to degludec. The DTR-QOL Factor 2, “Anxiety and dissatisfaction with treatment”, was significantly improved in patients with type 2 diabetes mellitus and especially in the subgroup receiving basal supported oral therapy (BOT).

**Conclusions:**

Switching of the basal insulin regimen from glargine or detemir to degludec significantly improved the QOL of patients with type 2 diabetes mellitus who were receiving BOT, by reducing mental stress or anxiety about their treatment.

## Background

Strict glycemic control is important for prevention of diabetic complications in patients with type 1 and type 2 diabetes mellitus. However, insulin therapies often have adverse effects on quality of life (QOL). Most patients with type 1 diabetes mellitus receive intensive insulin therapy. On the other hand, for patients with type 2 diabetes mellitus, basal supported oral therapy (BOT), consisting of basal insulin injection and an oral hypoglycemic agent, is widely used. Thus, long-acting insulin preparations play an important role in both types of patients. Three types of long-acting insulin are currently available, i.e., glargine, detemir, and degludec, which were introduced in 2000, 2002, and 2013, respectively. The duration of action of glargine is 27 h (range: 10.5–29.0) and that of detemir is 23 h (range: 4.0–30.0) [1], whereas degludec has a duration of action greater than 40 h, with a terminal half-life of more than 25 h [2]. Degludec is a slightly modified insulin that forms stable multi-hexamers; after injection of the preparation under subcutaneous tissue, monomeric degludec dissociates slowly from the ends of the multi-hexamers, resulting in a very long duration of action [3]. Degludec showed similar efficacy to glargine or detemir in terms of hemoglobin A1c (HbA1c) level and significantly reduced the risk of hypoglycemia during the night in both type 1 and type 2 diabetes mellitus patients in a large clinical trial, which included Japanese patients [4–6].

Despite the advantages of degludec, its effects on patients’ QOL have not yet been reported. In this work, we examined the change in QOL associated with a switch in basal insulin regimen from glargine or detemir to degludec in patients with type 1 and type 2 diabetes mellitus.

## Methods

### Study design and participants

Ten outpatients with type 1 and 20 outpatients with type 2 diabetes mellitus, who had not previously been treated with insulin degludec, were included in this prospective study at the Japan Community Healthcare Organization Kanazawa Hospital. The main inclusion criteria were age ≥ 20 years, diagnosis of type 1 or type 2 diabetes, treatment with glargine or detemir for at least 24 weeks before entry into this study, and less than 1.0 % HbA1c change during the 24 weeks before entry into this study. When we switched to degludec, injection was given once a day regardless of the number of injections (once or twice a day) of basal insulin. Written informed consent was obtained from each participant before any survey was carried out. Any patient who could not understand the QOL questionnaire was dropped from the study.

This study is a registered clinical study, number 13-02-01, and was conducted in accordance with the Declaration of Helsinki and Good Clinical Practice Guidelines. The protocol and the consent form were reviewed and approved by the local independent ethics committee of Japan Community Healthcare Organization, Kanazawa Hospital, before initiation of the study.

### Study objectives

Patient QOL was the primary outcome. All patients were evaluated using the Diabetes Therapy-Related QOL (DTR-QOL) scoring application (for iPad) developed by Ishii [7]. Patient-reported outcomes are divided into 4 factors: (1) Burden on social activities and daily activities, (2) Anxiety and dissatisfaction with treatment, (3) Hypoglycemia, and (4) Satisfaction with treatment. Factors 1, 2, 3, and 4 consist of 13, 8, 4, and 4 items, respectively (Table 1). The score of each item is rated using a 7-point Likert scale (1 is strongly agree, 7 is strongly disagree). The scores are converted to a range of 0–100, and the application automatically computes a total score. The DTR-QOL score correlates moderately well with Diabetes Treatment Satisfaction Questionnaire and MOS 8-Item Short-Form Health Survey scores [7]. In this study, we measured the DTR-QOL at baseline, and after 12 and 24 weeks of therapy with degludec.

**Table 1:** DTR-QOL items and domain structure

Factor 1: Burden on social activities and daily activities
1. My current diabetes treatment interferes with my work and activities.
2. My current diabetes treatment limits the scope of my activities.
3. It is difficult to find places on time for my current diabetes.
4. My current diabetes treatment interferes with group activities and personal friendships.
5. It is a burden getting up at a certain time every morning for my current diabetes treatment.
6. With my current diabetes treatment, the restricted meal times are a burden.
7. When I eat out, it is difficult to manage my current diabetes treatment.
8. I feel like my current diabetes treatment takes away the enjoyment of eating.
9. With my current diabetes treatment, it is hard to curb my appetite.
10. The time and effort to manage my current diabetes treatment are a burden.
11. I am constantly concerned about time to manage my current diabetes treatment.
12. Pain due to my current diabetes treatment is uncomfortable.
13. Gastrointestinal symptoms (nausea, passing gas, diarrhea, abdominal pain) due my current diabetes treatment are uncomfortable.
Factor 2: Anxiety and dissatisfaction with treatment
14. I am bothered by weight gain with my current diabetes treatment.
19. I have uncomfortable symptoms due to hyperglycemia (high blood glucose).
20. I am worried about high blood glucose.
21. I am dissatisfied that my blood glucose is unstable (high and low).
22. I am worried that complications might get worse with my current diabetes treatment.
23. I get anxious thinking about living while on my current diabetes treatment.
24. I find it unbearable to think that even if I continue my current diabetes treatment, my diabetes may not be cured.
25. I am concerned that if I continue my current diabetes treatment, the efficacy (effectiveness) may diminish.
Factor 3: Hypoglycemia
15. I worry about low blood glucose due to my current diabetes treatment.
16. I am scared because of low blood glucose.
17. I am sometimes bothered by low blood glucose.
18. Symptoms due to low blood glucose are uncomfortable.
Factor 4: Satisfaction with treatment
26. Overall, I am satisfied with my current blood sugar control (glycemic control).
27. With my current diabetes treatment, I am confident that I can maintain good blood glucose control.
28. I am hopeful about the future with my current diabetes treatment.
29. With regards to diabetes treatment, I am satisfied with current treatment methods.

Secondary outcomes included HbA1c, which was measured every 4 weeks and expressed as National Glycohemoglobin Standardization Program equivalent values [8]. Fasting blood glucose (FBG), 1,5-anhydroglucitol (1,5-AG), amount of insulin units taken and body weight were also evaluated at baseline and after 12 and 24 weeks of therapy. Baseline clinical characteristics of patients incorporated in this study were age, sex, body mass index (BMI), duration of diabetes mellitus, HbA1c, and prior insulin treatment, including oral hypoglycemic agents and frequency of injections (Table 2). In principle, the regimen was kept unchanged during this study.

**Table 2:** Baseline clinical characteristics of participants

Items	Type 1	Type 2
Number (male/female)	10 (5/5)	20 (11/9)
Age (years)	52.1 ± 15.3	61.9 ± 13.1
BMI (kg/m^2^)	22.2 ± 2.7	24.7 ± 4.8
Duration of diabetes (years)	7.5 ± 4.2	14.6 ± 7.6
HbA1c (%)	8.2 ± 1.4	7.6 ± 1.2
Prior treatment insulin		
Insulin glargine	8 (80 %)	13 (65 %)
Insulin detemir	2 (20 %)	7 (35 %)
Bolus insulin	10 (100 %)	7 (35 %)
The frequency of injections with basal insulin before switching		
Once	3 (30 %)	13 (65 %)
Twice	7 (70 %)	7 (35 %)
Other concomitant drugs		
Glucagon like peptide-1 receptor agonist	0 (0 %)	0 (0 %)
Sulphonylurea	0 (0 %)	2 (10 %)
Glinide	0 (0 %)	7 (35 %)
Metformin	0 (0 %)	10 (50 %)
Pioglitazone	0 (0 %)	2 (10 %)
α-glucosidase inhibitor	0 (0 %)	5 (25 %)
Dipeptidyl peptidase 4 inhibitor	0 (0 %)	8 (40 %)
Sodium glucose cotransporter 2 inhibitor	0 (0 %)	0 (0 %)

### Statistical analysis

All data were analyzed using IBM SPSS statistical software version 18 for Windows (SPSS Japan Inc., Tokyo, Japan). One-way analysis of variance (ANOVA) was used for comparisons of numerical values (DTR-QOL score, each item score, HbA1c, FBG, 1,5-AG, body weight and amount of insulin units between baseline and after treatment with degludec). Data were expressed as the mean ± standard deviation. P values under 5 % were considered to be significant.

## Results

### Changes of QOL after switching to degludec

**Fig. 1:**
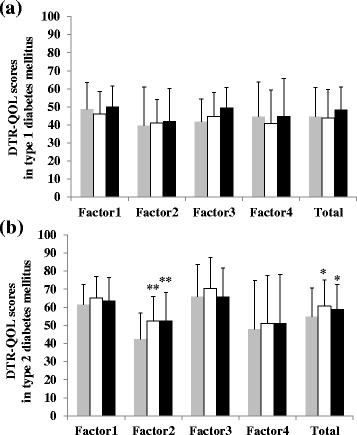
Changes of Factor and total scores in type 1 and type 2 diabetes mellitus. The upper figure (**a**) showed the type 1, the lower figure (**b**) shows the type 2 patients after switching from glargine or detemir to degludec. *Grey*, *white*, and *dark* columns indicate scores at baseline, 12 weeks, and 24 weeks, respectively. Data are mean ± SD (*bars*) for all assigned participants. Comparisons were made by ANOVA. ^*^ *p* < 0.05, ^**^ *p* < 0.005, significantly different from baseline

As shown in Fig. 1, patients with type 2 diabetes mellitus had higher total DTR-QOL scores than patients with type 1 diabetes mellitus throughout this study (baseline, 54.8 ± 11.3 points vs. 44.6 ± 11.6 points, *p* < 0.05; at 12 weeks, 60.7 ± 12.0 points vs. 43.8 ± 12.0 points, *p* < 0.005; at 24 weeks, 59.0 ± 12.9 points vs. 48.3 ± 11.2 points, *p* < 0.05). However, the DTR-QOL scores of patients with type 1 diabetes mellitus showed no significant change during degludec treatment (Fig. 1 (a)). On the other hand, the total DTR-QOL scores of patients with type 2 diabetes mellitus showed significant improvement (baseline, 54.8 ± 11.3 points; 12 weeks, 60.7 ± 12.0 points; 24 weeks, 59.0 ± 12.9 points; *p* < 0.05), as shown in Fig. 1 (b). Further, the DTR-QOL Factor 2 “Anxiety and dissatisfaction with treatment” subscale score was significantly improved in patients with type 2 diabetes mellitus at weeks 12 and 24 compared to baseline (baseline, 42.5 ± 18.0 points; 12 weeks, 52.5 ± 17.0 points; 24 weeks, 52.7 ± 14.0 points; *p* < 0.005) (Fig. 1 (b)).

**Fig. 2:**
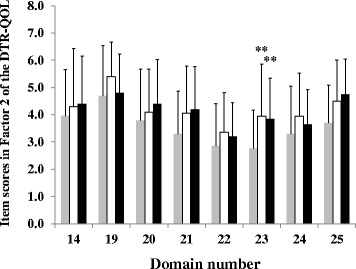
Changes of item scores in Factor 2 in patients with type 2 diabetes mellitus. *Grey*, *white*, and *dark* columns indicate item scores in Factor 2 at baseline, 12 weeks, and 24 weeks, respectively. Data are mean ± SD (*bars*) for all assigned participants. Comparisons were made by ANOVA. ^**^ *p* < 0.005, significantly different from baseline

Among the items within Factor 2, a significant improvement from baseline was seen in the score of item No. 23 “I get anxious thinking about living while on my current diabetes treatment” (baseline, 2.8 ± 1.4 points; 12 weeks, 4.0 ± 1.9 points; 24 weeks, 3.9 ± 1.5 points; *p* < 0.005) (Fig. 2).

**Fig. 3:**
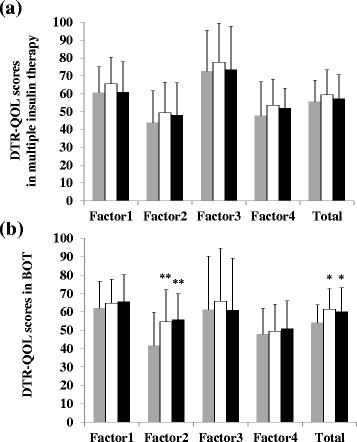
Changes of Factor and total scores in type 2 diabetes mellitus according to insulin treatment. The upper figure (**a**) shows the receiving multiple insulin therapy, the lower figure (**b**) shows the receiving BOT on type 2 patients. *Grey*, *white*, and *dark* columns indicate DTR-QOL score at baseline, 12 weeks, and 24 weeks, respectively. Data are mean ± SD (*bars*) for all assigned participants. Comparisons were made by ANOVA. ^*^ *p* < 0.05, ^**^ *p* < 0.005, significantly different from baseline

When we divided patients with type 2 diabetes mellitus into subgroups according to treatment, those who were receiving BOT (*n* = 13) showed significantly increased DTR-QOL scores (baseline, 54.3 ± 10.7 points; 12 weeks, 61.4 ± 11.1 points; 24 weeks, 60.2 ± 12.9 points; *p* < 0.05), whereas other patients (*n* = 7) did not (Fig. 3). Their Factor 2 scores were also significantly increased (baseline, 41.7 ± 18.0 points; 12 weeks, 54.7 ± 17.3 points; 24 weeks, 55.8 ± 14.1 points; *p* < 0.005) (Fig. 3). Moreover, their scores of item No. 23 showed significant improvement (baseline, 2.7 ± 1.4 points; 12 weeks, 4.1 ± 1.8 points; 24 weeks, 4.2 ± 1.7 points; *p* < 0.005).

### Glycemic control

**Fig. 4:**
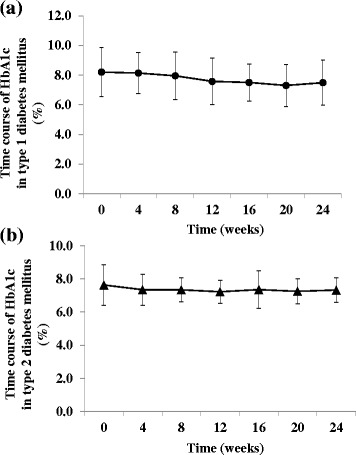
Time course of HbA1c in type 1 and type 2 diabetes mellitus patients. (**a**) HbA1c in type 1 patients (●) and (**b**) HbA1c in type 2 patients (▲) after switching from glargine or detemir to degludec. Data are mean ± SD (*bars*) for all assigned participants. Comparisons were made by ANOVA

FBG, 1.5-AG and HbA1c showed no significant change in either type 1 or type 2 diabetes mellitus patients during the study (Table 3 and Fig. 4).

**Table 3:** Change of secondary outcomes in participants

Term	Type	Baseline	12 weeks	24 weeks
FBG (mg/dL)	1	160.8 ± 68.7	150.2 ± 37.4	149.4 ± 46.7
	2	152.9 ± 61.5	141.1 ± 50.0	142.1 ± 51.5
1.5-AG (μg/mL)	1	6.9 ± 5.5	7.8 ± 6.5	9.7 ± 8.8
	2	8.0 ± 4.9	10.4 ± 7.2	10.4 ± 5.5
Bolus insulin (IU/day)	1	20.3 ± 6.5	19.4 ± 7.1	19.0 ± 7.3
	2	19.9 ± 5.7	20.1 ± 5.5	20.0 ± 5.6
Basal insulin (IU/day)	1	14.4 ± 5.1	12.3 ± 5.0 *	12.3 ± 6.1 *
	2	13.4 ± 7.6	11.9 ± 6.7 *	11.4 ± 6.3 *
Body weight (kg)	1	58.7 ± 11.4	57.9 ± 10.8	57.5 ± 11.1
	2	66.6 ± 14.7	66.8 ± 14.7	66.9 ± 14.6

Data are mean ± SD. Comparisons were made by ANOVA. ^*^ *p* < 0.05, significantly different from baseline

### Change in number of insulin units

The total number of bolus insulin units taken by patients with type 1 and type 2 diabetes mellitus did not change during the study (20.3 ± 6.5 units and 19.9 ± 5.7 units at baseline to 19.4 ± 7.1 units and 20.1 ± 5.5 units at week 12, 19.0 ± 7.3 units and 20.0 ± 5.6 units at week 24, respectively). However, the total number of basal insulin units in patients with type 1 and type 2 diabetes mellitus decreased significantly from 14.4 ± 5.1 unit and 13.4 ± 7.6 unit at baseline to 12.3 ± 5.0 unit and 11.9 ± 6.7 unit at 12 weeks, and to 12.3 ± 6.1 unit and 11.4 ± 6.3 unit at 24 weeks, respectively (Table 3).

### Change of body weight

Switching the basal insulin regimen to degludec had no significant effect on body weight of patients with either type 1 diabetes mellitus (58.7 ± 11.4 kg at baseline to 57.9 ± 10.8 kg at 12 weeks and 57.5 ± 11.1 kg at 24 weeks) or type 2 diabetes mellitus (66.6 ± 14.7 kg at baseline to 66.8 ± 14.7 kg at week 12 and 66.9 ± 14.6 kg at week 24) (Table 3).

### Occurrence of hypoglycemia

None of the patients with either type 1 or type 2 diabetes mellitus experienced serious hypoglycemia or reported blood sugar levels below 60 mg/dL (measured by self-monitoring).

## Discussion

Our results indicate that switching from glargine or detemir to degludec resulted in a significant improvement of the DTR-QOL score in patients with type 2 diabetes mellitus, but not in those with type 1 diabetes mellitus. DTR-QOL scores are associated with the levels of self-care activities [9], and we found that our type 2 patients showed a significant improvement in the score for Factor 2, “Anxiety and dissatisfaction with treatment”, which includes items dealing with weight gain, discomfort, anxiety, and dissatisfaction with treatment (Table 1). Specifically, we found a significant improvement in the score for item No. 23 “I get anxious thinking about living while on my current diabetes treatment”.

Previous reports have identified glycemic control, type of insulin therapy, and frequency of hypoglycemia as factors influencing QOL [9]. Therefore, we investigated the influence of these factors in our patients. Firstly, regarding glycemic control, HbA1c was measured every 4 weeks during the 24-week study, and we found no significant change in either type 1 or type 2 patients, in agreement with previous reports [4–6]. Further, there was no significant change in items related to hyperglycemia in Factor 2 of the DTR-QOL. These results suggest that the improvement of QOL cannot be explained in terms of lowering of HbA1c.

Secondly we investigated the relation between QOL and insulin treatment. Basal insulin units were decreased significantly in patients with both type 1 and type 2 diabetes mellitus (Table 3), but improvement of QOL was seen only in patients with type 2 diabetes mellitus, not in patients with type 1, suggesting that the decrease of insulin units cannot explain the improvement of QOL. It should be noted that 7 out of 10 patients with type 1 diabetes mellitus had received two injections of basal insulin and 3 injections of bolus insulin per day (total, 5 injections) during treatment with glargine or detemir, while switching to degludec reduced the number of basal injections to one (total, 4 injections). Glycemic control was well maintained, but it appeared that the reduction of injection frequency from 5 times to 4 times was not sufficient to significantly improve QOL of these patients. In the case of patients with type 2 diabetes mellitus, the number of injections per day remained the same after switching to degludec, even though the switch to degludec significantly improved QOL. Interestingly, subgroup analysis according to type of treatment revealed a significant QOL improvement in patients receiving BOT (13 out of 20 patients) (Fig. 3). There was no significant difference in the clinical background between patients receiving BOT and other patients with type 2 diabetes mellitus. These results suggest that the key factor for QOL improvement is the difference of corrective action if an injection is forgotten. For pharmacokinetic reasons [10], doctors at our hospital advise that if patients with type 1 and type 2 diabetes mellitus forget to take an injection of glargine or detemir for more than 2 h after the scheduled time, they should not self-medicate to avoid hypoglycemia, but should contact their doctors to ask for instructions. On the other hand, if patients taking degludec forget an injection, they can take it when they remember, and then take the next injection at least eight hours later, since the daily injection time of degludec can be varied without compromising glycemic control or safety because of its long duration of action [11]. This flexibility may be especially beneficial in BOT therapy, since degludec can be injected at the patient’s convenience. Therefore, we speculate that the improvement of QOL in type 2 patients was due to a decrease of mental stress associated with treatment, because of the flexibility in timing of degludec injection. This is consistent with the improvement in the score of item No. 23 in Factor 2 of the DTR-QOL (“I get anxious thinking about living while on my current diabetes treatment”). The validity of the improvement in the score of item No. 23 is also supported by the observation of improvements of 0.3–0.8 points from baseline in the score of items No.1, No.2, No.3, No.6, No.7 and No.11 in Factor 1 of the DTR-QOL, though these did not reach statistical significance. On the other hand, patients with type 1 diabetes mellitus are well aware that insulin therapy is essential for life support, and basal insulin is often administered simultaneously with bolus insulin, so these patients may not feel the benefits of flexible injection of degludec.

Finally, we found no improvement of Factor 3, “Hypoglycemia” in this study, even though degludec is reported to significantly reduce the frequency of hypoglycemia during the night, compared to glargine or detemir [4–6]. The reason for this may be that our target patients had not experienced hypoglycemia while receiving glargine or detemir, and also did not experience it after switching to degludec, so that there was no change of QOL in this respect.

The present study has several limitations. The number of cases was small, because this research was performed at a single site. Also, this exploratory study used an open label design. Further, insulin degludec was administered with a new device, Flextouch® [12–14]. There were differing opinions, both positive and negative, about the new device in our survey. In the future, we need to clarify the influence of the delivery device of insulin therapy on QOL.

## Conclusions

An important finding in our study was that switching to degludec from glargine or detemir in patients with type 2 diabetes mellitus, especially those who were receiving BOT, improved the QOL, even though there was no significant change of HbA1c. This improvement of QOL may be attributed to a reduction of mental stress and anxiety due to the greater flexibility of treatment timing with degludec.

## Abbreviations

QOL: quality of life
HbA1c: hemoglobin A1c
DTR-QOL: Diabetes Therapy-Related QOL
BOT: basal supported oral therapy
FBG: fasting blood glucose
1,5-AG: 1,5-anhydroglucitol
BMI: body mass index
